# Initiation of the SGLT2 inhibitor canagliflozin to prevent kidney and heart failure outcomes guided by HbA1c, albuminuria, and predicted risk of kidney failure

**DOI:** 10.1186/s12933-022-01619-0

**Published:** 2022-09-23

**Authors:** Sok Cin Tye, Niels Jongs, Steven G. Coca, Johan Sundström, Clare Arnott, Bruce Neal, Vlado Perkovic, Kenneth W. Mahaffey, Priya Vart, Hiddo. J. L. Heerspink

**Affiliations:** 1 Department of Clinical Pharmacy and Pharmacology, University of Groningen, University Medical Center Groningen, Hanzeplein 1, PO Box 30001, 9700 RB Groningen, The Netherlands; 2 Division of Nephrology, Department of Medicine, Icahn School of Medicine at Mount Sinai, New York, NY USA; 3 Department of Medical Sciences, Uppsala University, Uppsala, Sweden; 4 The George Institute for Global Health, UNSW Sydney, Sydney, NSW Australia; 5 Department of Cardiology, Royal Prince Alfred Hospital, Sydney, Australia; 6 Stanford Center for Clinical Research, Department of Medicine, Stanford University, Stanford, CA USA

**Keywords:** Canagliflozin, Type 2 diabetes, Treatment strategy, Kidney disease, Heart failure

## Abstract

**Background:**

Sodium glucose co-transporter-2 (SGLT2) inhibitors reduce the risk of kidney and heart failure events independent of glycemic effects. We assessed whether initiation of the SGLT2 inhibitor canagliflozin guided by multivariable predicted risk based on clinical characteristics and novel biomarkers is more efficient to prevent clinical outcomes compared to a strategy guided by HbA1c or urinary-albumin-creatinine ratio (UACR) alone.

**Methods:**

We performed a post-hoc analysis of the CANVAS trial including 3713 patients with available biomarker measurements. We compared the number of composite kidney (defined as a sustained 40% decline in eGFR, chronic dialysis, kidney transplantation, or kidney death) and composite heart failure outcomes (defined as heart failure hospitalization or cardiovascular (CV) death) prevented per 1000 patients treated for 5 years when canagliflozin was initiated in patients according to HbA1c ≥ 7.5%, UACR, or multivariable risk models consisting of: (1) clinical characteristics, or (2) clinical characteristics and novel biomarkers. Differences in the rates of events prevented between strategies were tested by Chi^2^-statistic.

**Results:**

After a median follow-up of 6.1 years, 144 kidney events were recorded. The final clinical model included age, previous history of CV disease, systolic blood pressure, UACR, hemoglobin, body weight, albumin, estimated glomerular filtration rate, and randomized treatment assignment. The combined biomarkers model included all clinical characteristics, tumor necrosis factor receptor-1, kidney injury molecule-1, matrix metallopeptidase-7 and interleukin-6. Treating all patients with HbA1c ≥ 7.5% (n = 2809) would prevent 33.0 (95% CI 18.8 to 43.3 ) kidney events at a rate of 9.6 (95% CI 5.5 to 12.6) events prevented per 1000 patients treated for 5 years. The corresponding rates were 5.8 (95% CI 3.4 to 7.9), 16.6 (95% CI 9.5 to 22.0) (P < 0.001 versus HbA1c or UACR approach), and 17.5 (95% CI 10.0 to 23.0) (P < 0.001 versus HbA1c or UACR approach; P = 0.54 versus clinical model). Findings were similar for the heart failure outcome.

**Conclusion:**

Initiation of canagliflozin based on an estimated risk-based approach prevented more kidney and heart failure outcomes compared to a strategy based on HbA1c or UACR alone. There was no apparent gain from adding novel biomarkers to the clinical risk model. These findings support the use of risk-based assessment using clinical markers to guide initiation of SGLT2 inhibitors in patients with type 2 diabetes.

**Supplementary Information:**

The online version contains supplementary material available at 10.1186/s12933-022-01619-0.

## Introduction

Sodium glucose cotransporter-2 (SGLT2) inhibitors are developed and approved by regulatory agencies for the treatment of type 2 diabetes to improve glycemic control. Following regulatory requirements to establish cardiovascular (CV) safety of new anti-diabetic treatments, the CV safety of SGLT2 inhibitors was assessed in large clinical trials [1, 2]. These trials demonstrated that these agents markedly reduce the risk of kidney and heart failure outcome in patients with type 2 diabetes. Subsequent trials also demonstrated the efficacy and safety of these agents to prevent kidney and heart failure outcomes in patients with chronic kidney disease (CKD) or heart failure without type 2 diabetes [3–6]. Clinical practice guidelines recommend the use of SGLT2 inhibitors in patients with type 2 diabetes and atherosclerotic CV disease, CKD, or heart failure as second-line treatment on top of metformin [7, 8]. Despite the efficacy of SGLT2 inhibitors in patients with and without type 2 diabetes, the initiation of these agents in clinical practice is slow and treatment is centered around isolated risk marker improvement such as glycated hemoglobin (HbA1c) or albuminuria [9–11].

Analyses from CV and kidney outcome trials have demonstrated that the relative benefits of SGLT2 inhibitors are consistent across many patient subgroups including those with or without type 2 diabetes, severity of kidney disease or the presence of heart failure [12, 13]. The absolute benefits however, are greater as baseline risk increases, such as in those with low estimated glomerular filtration rate (eGFR) or high albuminuria [14, 15]. This suggests that a treatment approach guided by the individual risk of each participant, as determined by the presence of kidney or CV risk markers, would be more efficient for preventing clinical outcomes compared to a treatment strategy based on HbA1c or albuminuria alone.

In this study, we first assessed if a treatment strategy to initiate the SGLT2 inhibitor canagliflozin using the multivariable predicted risk of kidney outcome based on clinical risk markers is more efficient for preventing clinical outcomes compared to a strategy based on HbA1c or albuminuria alone. Secondly, we assessed whether the addition of novel biomarkers to predict kidney outcome further improve the efficiency of guiding SGLT2 inhibitor initiation. Because of shared pathophysiology between diabetic kidney disease and heart failure, we hypothesized that the kidney risk model would also stratify patients at risk for a clinically relevant heart failure event and therefore also report the effects of canagliflozin on heart failure outcome by baseline kidney risk.

## Methods

### Study design and participants

This study included data from the Canagliflozin Cardiovascular Assessment Study (CANVAS) program consisting of two multicentre, randomized, double-blind placebo-controlled trials (CANVAS and CANVAS-R) to assess the CV safety and kidney outcomes of canagliflozin in type 2 diabetes patients with an established CV disease or multiple CV risk factors. The CANVAS trial included a total of 4330 patients who were randomly assigned to canagliflozin 100 mg, 300 mg, or placebo in a 1:1:1 ratio, and the CANVAS-R included 5812 patients. The inclusion criteria for the CANVAS program were patients with type 2 diabetes (glycated hemoglobin level, ≥ 7.0% and ≤ 10.5%), either 30 years of age or older with a history of symptomatic atherosclerotic CV disease, or 50 years of age or older with two or more of the following risk factors for CV disease; diabetes duration more than 10 years, systolic blood pressure (BP) > 140 mmHg while receiving one or more anti-hypertensive agents, current smoker, evidence of micro- or macroalbuminuria, high-density lipoprotein (HDL) cholesterol level < 1mmol/L, and eGFR of  ≥ 30 mL/min/1/.73m^2^ at baseline. For additional exploratory biomarker research, CANVAS patients provided separate informed consent form (excluding CANVAS-R patients). The CANVAS trial was conducted in accordance with the Declaration of Helsinki (clinicaltrials.gov: NCT01032629). The study protocol was approved by ethics committee at each participating site. All patients provided written informed consent before study specific procedures commenced [16].

### Biomarker measurement

Blood samples of eligible patients were measured at baseline. Plasma tumor necrosis factor receptor-1 (TNFR-1), TNFR-2, and kidney injury molecule-1 (KIM-1) were measured using the Mesoscale Quickplex SQ 120 platform (Meso Scale Diagnostics [MSD]), a high-performance electrochemiluminescence immunoassay (Analyses performed by RenalytixAI, New York, NY, USA). Plasma Growth Differentiation Factor-15 (GDF-15) was measured using the Elecsys^®^ GDF-15 electrochemiluminescence immunoassay (Roche Diagnostics International Ltd, Rotkreuz, Switzerland). Plasma interleukin-6 (IL-6), serum Matrix Metallopeptidase-7 (MMP-7), urinary monocyte chemoattractant protein-1 (uMCP-1), and urinary epidermal growth factor (uEGF) were measured using the Mesoscale QuickPlex SQ 120 platform (MSD, Rockville, MD, USA). All urinary measurements were standardized for urinary creatinine to correct for hydration status.

### Statistical analysis

#### Model derivation and validation

For model development, Cox proportional hazards regression models were used for prediction of the 5-year risk and treatment effect of canagliflozin on the composite kidney outcome (defined as a sustained 40% decline in eGFR, end-stage kidney disease with eGFR < 15 mL/min/1/.73m^2^, or the need of dialysis, kidney transplantation, or kidney death) and the composite heart failure outcome (defined as heart failure hospitalization or CV death).

Two risk prediction models were developed. First, a model based on clinical markers including the following candidate predictors: age, sex, race, smoking status, history of cardiovascular disease, systolic BP, HbA1c, urinary-albumin-to-creatinine-ratio (UACR), hemoglobin, body weight, HDL cholesterol, low-density-lipoprotein cholesterol (LDL), uric acid, potassium, eGFR according to the Chronic Kidney Disease Epidemiology Collaboration (CKD-EPI) 2009 equation, phosphorus, albumin level, and canagliflozin treatment (yes/no). Second, we added to the clinical model the following novel biomarkers as candidate predictors: TNFR-1, KIM-1, GDF-15, MMP-7, IL-6, uMCP-1 to creatinine ratio (uMCP-1/Cr) and uEGF to creatinine ratio (uEGF/Cr). To prevent overfitting, variables were selected based on a stepwise backward selection according to the Akaike’s Information Criterion [17]. We developed the prediction model for kidney outcomes from the linear combination of covariates from the final Cox proportional hazards regression model. We used the same variables included in the prediction model for kidney outcome to predict the heart failure outcome.

We tested the Cox proportional hazards assumptions using Schoenfeld residuals against log transformed time. The assumption was met for all included variables. We determined the Martingale distribution to visually inspect the linearity of the continuous variables, leading to log transformation of UACR, TNFR1, KIM-1, GDF-15, MMP-7, IL-6, uMCP-1/Cr and uEGF/Cr. To determine the internal validity of the final models, we generated 1000 bootstrap samples of the CANVAS dataset [18]. Calibration, describing the agreement between predicted probabilities and actual observed event rates, was demonstrated by calibration plots and chi-square goodness of fit tests [19]. The calibration plots were based on deciles of predicted risk to enable enough patients in each group. Discrimination, referring to the ability of each model to distinguish individuals who had an event versus those who did not, was quantified using the C-statistic. The 95% confidence limits (CI) of the C-statistic were calculated using a bootstrap procedure with 1000 replications using the percentile method [20].

To account for missing data, multiple imputations by chained equation (using the R package “MICE”, version 3.11.0) was performed on all variables that had missing values. Imputations were performed by a predictive mean matching, a semi-parametric approach which replaces missing values according to a multivariable regression [21]. Means and standard deviations are provided for variables with a normal distribution. For UACR, TNFR1, TNFR2, KIM1, GDF15, MMP-7, IL-6, uMCP-1/ Cr, and uEGF/ Cr, medians and interquartile ranges are reported due to their non-parametric distribution.

#### Individual baseline risk and treatment effect estimation

The 5-year predicted risk for the composite kidney outcome for all patients was calculated by setting the treatment status to zero in the development model (irrespective of patient’s actual treatment allocation). The on-treatment risk (OTR, %) was estimated by applying the treatment efficacy estimate to the predicted 5-year risk of an individual for each endpoint in the CANVAS trial. The absolute risk reduction (ARR) (%) for every individual (i.e., individual treatment effect) was calculated as the difference between the predicted 5-year baseline risk and the on-treatment risk. The formula to compute predicted baseline risk according to multivariable risk strategies and OTR are provided in Additional file 1: Box S1. Heterogeneity of treatment effect across baseline risk was assessed by adding interaction term between the predicted baseline risk and treatment (treatment * baseline risk) for the composite kidney and composite heart failure outcomes respectively.

#### Numbers needed to treat, and events prevented

To assess the efficacy of different treatment strategy, we ranked all patients by descending levels of baseline risk according to the HbA1c or UACR cut-offs, or the risk percentiles according to the clinical characteristics or the clinical/ novel biomarkers model. For each strategy, we assume that everyone above the threshold is treated and those below would be untreated. The number needed to treat (NNT) at a given threshold was estimated by using the reciprocal of the ARR at that threshold, with 95% CI calculated based on lower and upper limit of the original point estimate to account for statistical uncertainty. We assessed and compared the performance of each strategy at a given risk threshold by comparing the number of events prevented per 1000 patients treated for 5 years using Chi^2^-statistics. In addition, we performed an analysis by comparing the events prevented and NNT in participants with UACR ≥ 30 mg/g for the HbA1c versus UACR or the two multivariable risk-based strategies. Two-sided P-values < 0.05 were considered statistically significant. All statistical analyses were conducted with R version 4.1.1 (R Project for Statistical Computing, http://www.r-project.org).

## Results

### Baseline characteristics and clinical outcomes

A total of 4330 patients were included in the CANVAS trial of whom 3713 (85.8%) patients had biomarkers measured at baseline. The mean age was 62.7 years and 67.0% were males. (Table 1) At baseline, 59.4% of the patients had a history of atherosclerotic CV disease. Mean baseline eGFR was 79.5 ml/min/1.73m^2^ and median UACR was 11.7 mg/g. Demographic and clinical characteristics were balanced between the placebo and the treatment group. During a median follow-up of 6.1 years, 144 (3.9%) patients developed a composite kidney outcome, and 371 (10.0%) patients experienced a composite heart failure outcome.

**Table 1:** Baseline characteristics of patients included in the CANVAS Trial

Description	Canagliflozin (N = 2472)	Placebo (N = 1241)	Total (N = 3713)
Age (years)	62.8 (7.9)	62.6 (7.8)	62.7 (7.9)
Males, N (%)	1653 (66.9)	833 (67.1)	2486 (67.0)
Race, N (%)			
Caucasian	1992 (80.6)	997 (80.3)	2989 (80.5)
Black	60 (2.4)	32 (2.6)	92 (2.5)
Asian	257 (10.4)	134 (10.8)	391 (10.5)
Others	163 (6.6)	78 (6.3)	241 (6.5)
Smoker (yes)	429 (17.4)	250 (20.1)	679 (18.3)
Previous CVD history (yes)	1474 (59.6)	730 (58.8)	2204 (59.4)
eGFR (ml/min/1.73m2)	79.6 (17.4)	79.4 (17.3)	79.5 (17.3)
Glycated hemoglobin (%)	8.2 (0.9)	8.2 (0.9)	8.2 (0.9)
Systolic BP (mmHg)	136.3 (15.9)	137.2 (15.8)	136.6 (15.9)
UACR (mg/g)	11.7 [6.5 to 35.0]	11.6 [6.3 to 36.5]	11.7 [6.4 to 35.7]
Weight (kg)	93.6 (20.9)	92.9 (20.7)	93.4 (20.8)
Hemoglobin (g/L)	140.6 (14.5)	140.0 (14.4)	140.4 (14.5)
Albumin (g/L)	41.0 (3.3)	41.0 (3.2)	41.0 (3.2)
HDL-cholesterol (mmol/L)	1.2 (0.3)	1.2 (0.3)	1.2 (0.3)
LDL-cholesterol (mmol/L)	2.3 (0.9)	2.3 (0.9)	2.3 (0.9)
Uric acid (umol/L)	351.5 (91.8)	350.5 (98.5)	351.2 (94.1)
Potassium (mmol/L)	4.4 (0.5)	4.4 (0.4)	4.4 (0.5)
TNFR-1 (ng/ml)	2.6 [2.1 to 3.2]	2.6 [2.1 to 3.1]	2.6 [2.1 to 3.2]
TNFR-2 (ng/ml)	9.7 [7.8 to 12.0]	9.6 [7.9 to 12.1]	9.7 [7.9 to 12.0]
KIM-1 (ng/ml)	0.1 [0.1 to 0.2]	0.1 [0.1 to 0.2]	0.1 [0.1 to 0.2]
GDF-15 (ng/ml)	1.8 [1.2 to 2.5]	1.8 [1.2 to 2.5]	1.8 [1.2 to 2.5]
MMP-7 (ng/ml)	6.1 [4.7 to 8.4]	6.2 [4.7 to 8.4]	6.2 [4.7 to 8.4]
IL-6 (pg/ml)	1.6 [1.2 to 2.4]	1.6 [1.2 to 2.3]	1.6 [1.2 to 2.4]
uMCP-1 (ng/ml)	0.2 [0.1 to 0.3]	0.2 [0.1 to 0.3]	0.2 [0.1 to 0.3]
uEGF (ng/ml)	4.6 [2.6 to 7.8]	4.6 [2.6 to 7.9]	4.6 [2.6 to 7.8]
uMCP-1/Cr (ng/g)	191.0 [121.8 to 312.7]	193.0 [123.0 to 344.1]	191.7 [122.6 to 321.1]
uEGF/Cr (ng/g)	5063.3 [3345.4 to 7865.9]	5198.9 [3450.0 to 7658.5]	5097.4 [3365.7 to 7811.7]

For numerical variables which are normally distributed, data is presented as mean (SD). For UACR, TNFR-1, TNFR-2, KIM-1, GDF-15, MMP-7, IL-6, uMCP-1,uEGF, uMCP-1/Cr, uEGF/Cr with a skewed distribution, median [IQR] is presented. Categorical variables are presented as frequency (%)

Estimated glomerular filtration rate (eGFR) was estimated according to the Chronic Kidney Disease Epidemiology Collaboration formula as per the CANVAS trial protocol

BP, blood pressure; UACR, urinary-albumin to urinary-creatinine ratio; HDL, high-density-lipoprotein; LDL, low-density-lipoprotein.; TNFR, tumor necrosis factor receptor; KIM, kidney injury molecule; GDF, growth differentiation factor; MMP, matrix metallopeptidase; IL, interleukin; uMCP, urinary monocyte chemoattractant protein; uEGF, urinary epidermal growth factor; uMCP-1/Cr, urinary monocyte chemoattractant protein-1 to urinary creatinine ratio; uEGF/Cr, urinary epidermal growth factor to urinary creatinine ratio

### Model derivation and validation

**Fig. 1:**
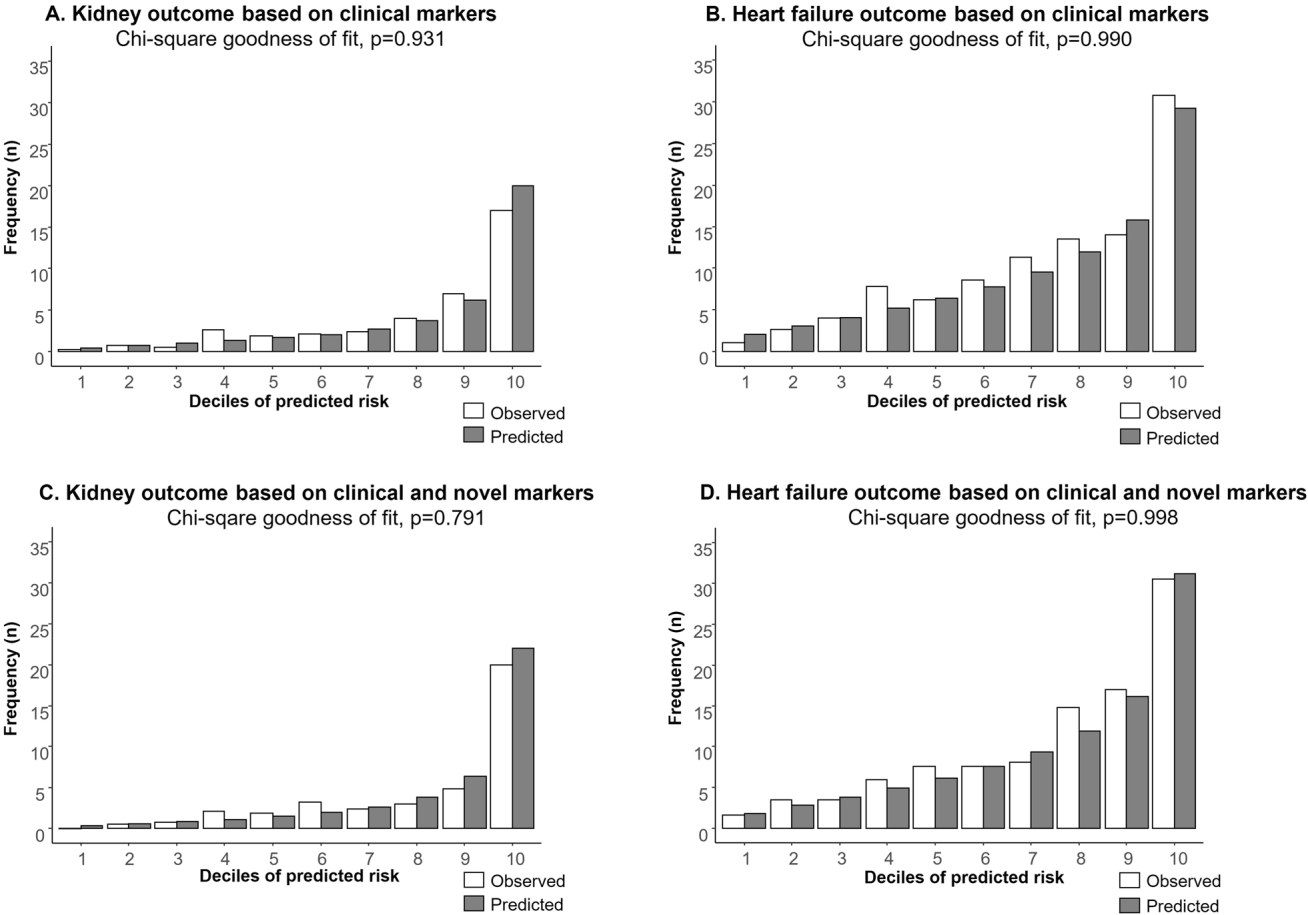
(Top) Calibration plots for the observed and predicted risk according to clinical markers, for **A** composite kidney (defined as the composite of sustained 40% decline of eGFR, end-stage kidney disease with eGFR < 15 mL/min/1.73 m², or need for dialysis or kidney transplantation, or kidney death); and **B** composite heart failure outcome in the CANVAS trial. (Bottom) Calibration plots for the observed and predicted risk according to clinical and novel biomarkers, for the **C** composite kidney and **D** composite heart failure outcome in the CANVAS trial

The final clinical model for the prediction of the kidney outcome included age, previous history of atherosclerotic CV disease, systolic BP, UACR, hemoglobin, body weight, albumin, eGFR, and the treatment variable. The bootstrap validation of the clinical model to predict the kidney outcome showed calibration with Chi^2^-goodness of fit test, P = 0.931 and good discrimination with C-statistics 0.80 (95% CI 0.75 to 0.84). For the composite heart failure outcome, the bootstrapped calibration showed Chi^2^-goodness of fit, P = 0.990 and C-statistics for discrimination was moderately good 0.73 (95% CI 0.70 to 0.76) (Fig. 1A and B).

Adding novel biomarkers to the clinical risk prediction model, TNFR1, KIM-1, MMP-7 and IL-6 were retained after backward selection for prediction of the composite kidney outcome. The calibration plots and Chi^2^-goodness of fit test indicated that the observed and predicted probability of the composite kidney and heart failure outcomes did not differ (P = 0.791 and P = 0.998; respectively) (Fig. 1C and D). The discrimination index to predict the 5-year composite kidney risk was good [C-statistics 0.81 (95% CI 0.76 to 0.86; difference versus clinical model 0.01 (95% CI − 0.01 to 0.04)], and moderately good for the composite heart failure outcome [C-statistics 0.74 (95% CI 0.71 to 0.77; difference versus clinical model 0.01 (95% CI 0.00 to 0.02)].

### 5-year risk prediction and absolute risk reductions of the composite kidney and the composite heart failure outcome

**Fig. 2:**
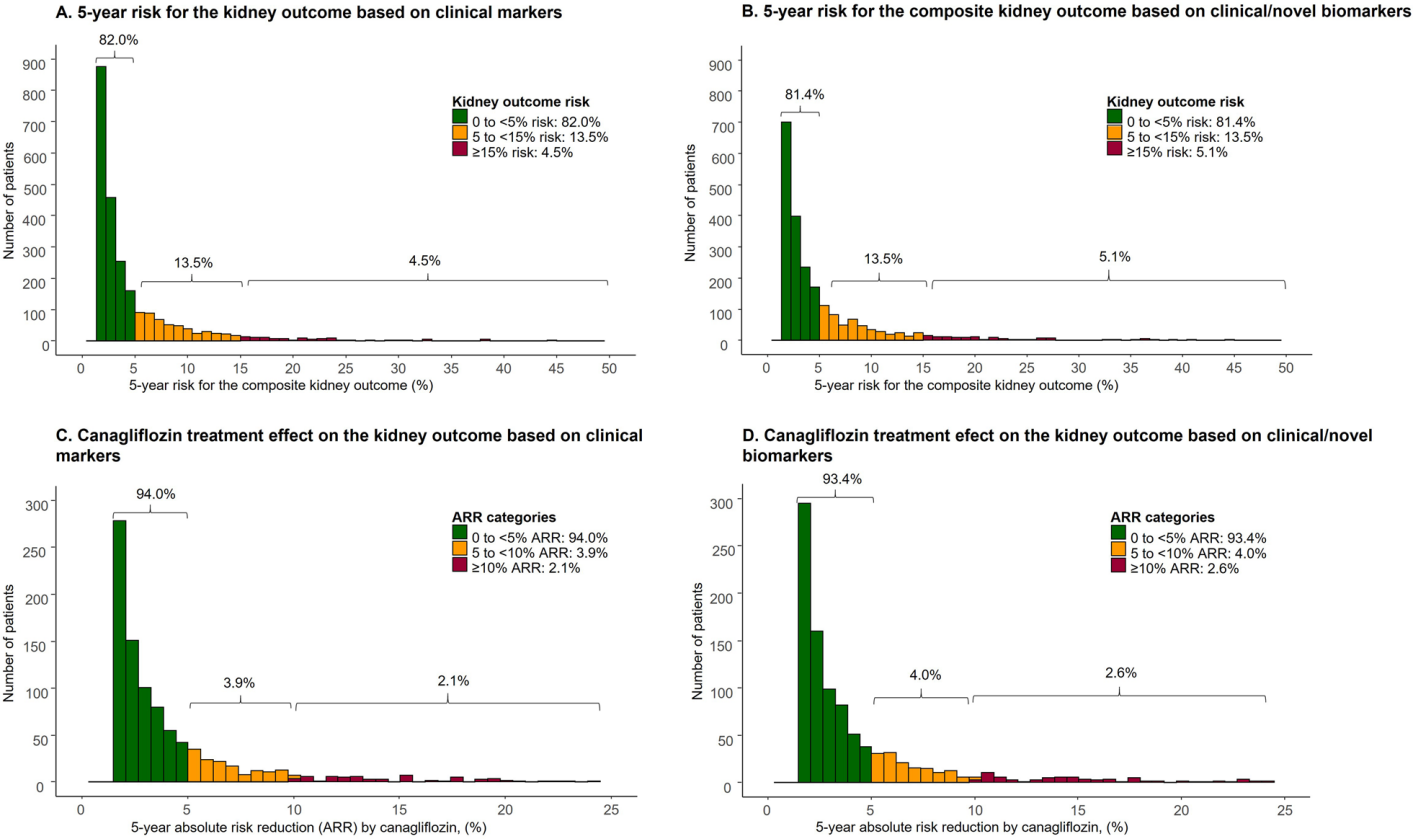
(Top) The distribution of predicted 5-year risk in patients with type 2 diabetes for the composite kidney outcome (Defined as a composite of sustained 40% decline in eGFR, end-stage kidney disease [defined as an eGFR < 15 mL/min/1/.73m^2^] or the need of dialysis, kidney transplantation, or kidney death) according to the **A** clinical markers and **B** clinical and novel biomarkers model. (Bottom) The distribution of individual treatment effect of canagliflozin on the predicted 5-year risk for the composite kidney outcome in patients with type 2 diabetes in the CANVAS trial according to the **C** clinical markers and **D** clinical and novel biomarkers model. Treatment effect is expressed as absolute risk reduction (ARR).

**Fig. 3:**
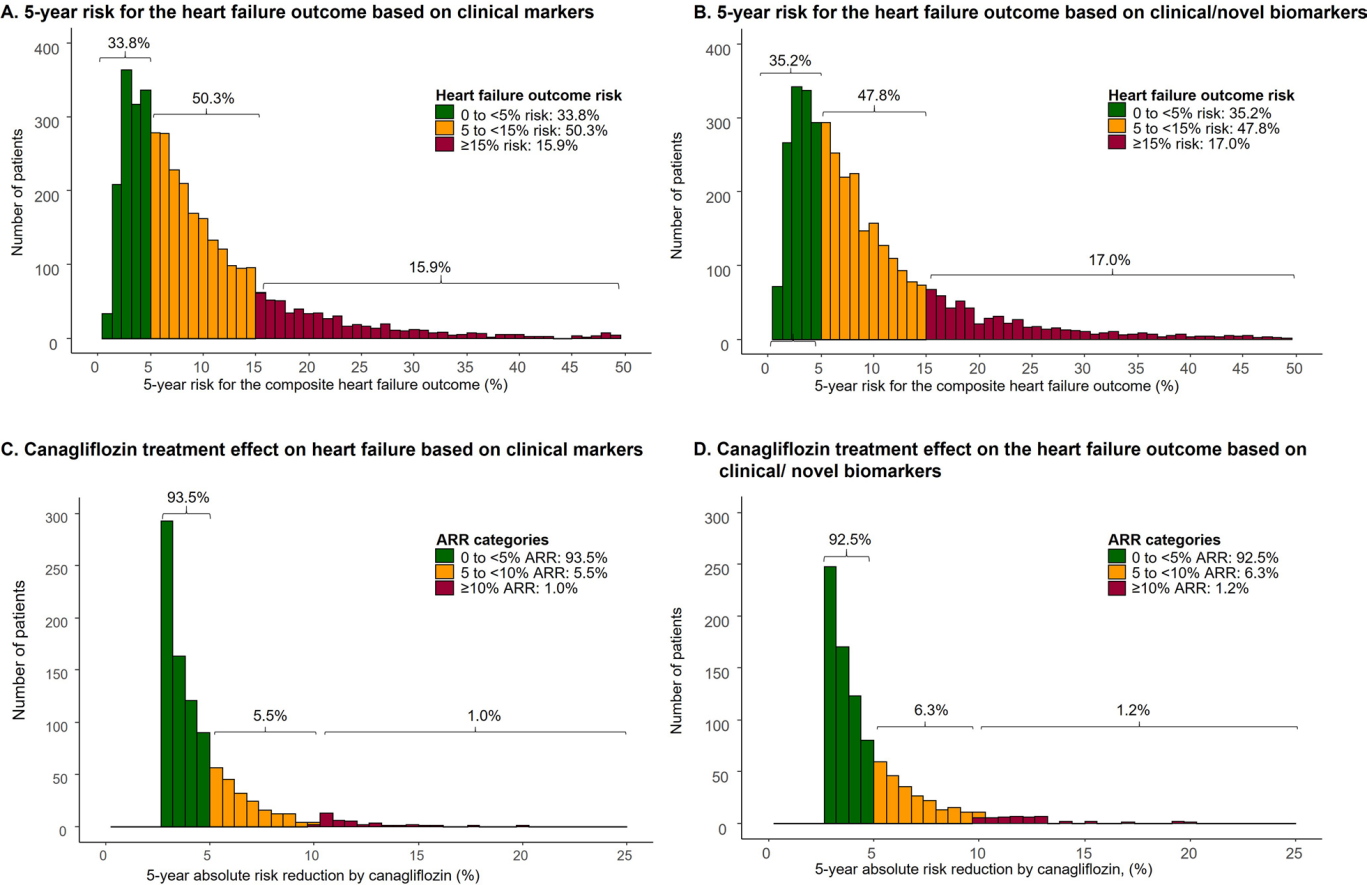
(Top) The distribution of predicted 5-year risk in type 2 diabetes patients for the composite of heart failure outcome (defined as heart failure hospitalization or CV death) according to the **A** clinical markers and **B** clinical and novel biomarkers model. (Bottom) The distribution of individual treatment effect of canagliflozin on the predicted 5-year risk for the composite of heart failure outcome in type 2 diabetes patients in the CANVAS trial according to the **C** clinical markers and **D** clinical and novel biomarkers model. Treatment effect is expressed as absolute risk reduction (ARR)

There was a wide distribution in the predicted risk between individuals for the composite kidney (Fig. 2A and B) and the composite heart failure (Fig. 3A and B) outcomes. Approximately 18% of patients had a predicted 5-year composite kidney risk higher than 5%, and about 65% of the patients had a predicted 5-year risk composite heart failure risk above 5%. There was also a large variation in terms of the absolute treatment benefit for the composite kidney (Fig. 2C and D) and the composite heart failure outcome (Fig. 3C and D). No heterogeneity of proportional treatment effect was observed across baseline risk for the composite kidney (P for interaction = 0.106 and P for interaction = 0.903) and the composite heart failure outcome (P for interaction = 0.561 and P for interaction = 0.750) in both clinical and novel biomarkers models, respectively (Additional file 1: Table S1).

### Numbers needed to treat, and events prevented

**Fig. 4:**
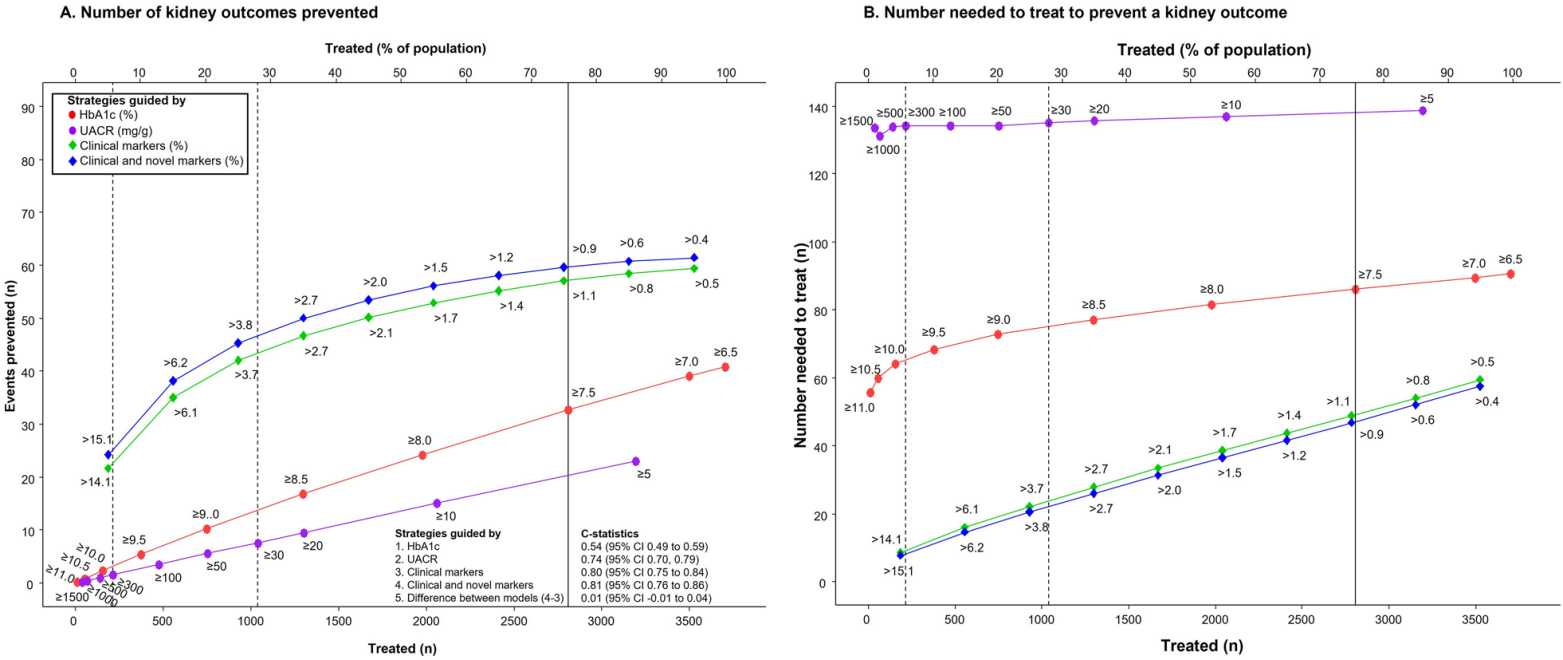
**A** The number of events prevented based on the HbA1c (red line), urinary-albumin-creatinine ratio (UACR) (purple line) clinical markers (green line), or clinical and novel biomarkers (blue line) strategy for the composite kidney outcome (defined as the composite of sustained 40% decline of eGFR, end-stage kidney disease with eGFR < 15 mL/min/1.73 m², or need for dialysis or kidney transplantation, or kidney death) outcome, and C-statistics obtained for the respective model. Numbers at each curve are specific HbA1c or UACR cut-offs, or based on 5–95th percentiles of predicted 5-year risk at specific treatment threshold. **B** The number needed to treat in order to avoid one composite kidney outcome according to the HbA1c (red line), UACR (purple), clinical markers (green line) or the clinical and novel markers (blue line) strategies are shown in the same figure. The intersection points at the vertical solid-line compares the number of events prevented or the number needed to treat (NNT) when 2809 patients are treated according to the HbA1c approach. The intersection points at the vertical dashed-lines compare the number of events prevented or the NNT when patients are treated according to UACR ≥ 30 mg/g (n = 1037) or UACR ≥ 300 mg/g (n = 214)

**Fig. 5:**
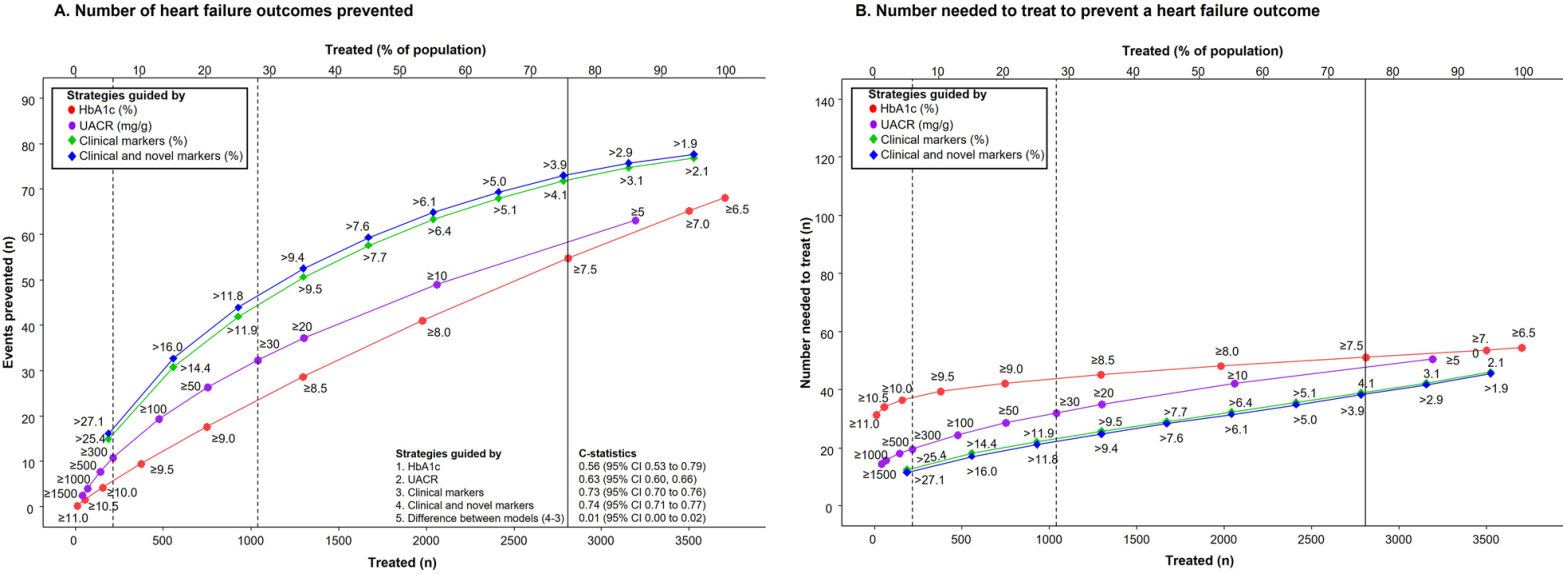
**A** The number of events prevented based on the HbA1c (red line), urinary-albumin-creatinine ratio (UACR) (purple line), clinical markers (green line), or clinical and novel biomarkers (blue line) strategy for the composite heart failure outcome (defined as heart failure hospitalization or CV death) outcome, and C-statistics obtained for the respective model. Numbers at each curve are specific HbA1c cut-offs or based on 5–95th percentiles of predicted 5-year risk at specific treatment threshold. **B** The number needed to treat in order to avoid one composite heart failure outcome according to the HbA1c (red line), clinical markers (green line) or the clinical and novel markers (blue line) strategies are shown in the same figure. The intersection points at the vertical solid-line compares the number of events prevented or the number needed to treat (NNT) when 2809 patients are treated according to the HbA1c approach. The intersection points at the vertical dashed-lines compare the number of events prevented or the NNT when patients are treated according to UACR ≥ 30 mg/g (n = 1037) or UACR ≥ 300 mg/g (n = 214).

The strategy to initiate SGLT2 inhibitors in patients with type 2 diabetes according to HbA1c ≥ 7.5% would involve treating 2809 (78.3%) of the population in order to prevent 33.0 (95% CI 18.8 to 43.3) kidney events. Treating 2809 patients according to the UACR cut-offs, clinical or clinical/ novel biomarker approach resulted in the prevention of 20.0 (95% CI 11.6 to 27.2), 57.0 (95% CI 32.7 to 75.4) and 60.0 (95% CI 34.2 to 78.7) kidney events (Fig. 4A). For the composite heart failure outcome, treating 2809 patients with HbA1c ≥ 7.5% would prevent 55.0 (95% CI 22.4 to 82.2) events as compared to 58.0 (95% CI 23.5 to 87.3), 72.0 (95% CI 29.3 to 107.6) and 73.0 (95% CI 29.8 to 109.4) events prevented when using the UACR, clinical or clinical/novel biomarker approach, respectively. (Fig. 5A) The results of the efficacy of different treatment strategies to prevent composite kidney or heart failure outcomes among patients with UACR ≥ 30 mg/g (n = 1037) are shown in Additional file 1: Figures S1A and S2A.

The NNT to prevent one composite kidney outcome according to HbA1c ≥ 7.5, UACR, or the two multivariable risk strategies were 86.0 (95% CI 64.9 to 149.5), 134 (95% CI 100.9 to 233.8), 49.0 (95% CI 37.3 to 85.9), and 47.0 (95% CI 35.7 to 82.2), respectively (Fig. 4B). This corresponded to 9.6 (95% CI 5.5 to 12.6), 5.8 (95% CI 3.4 to 7.9),16.6 (95% CI 9.5 to 22.0) (P < 0.001 vs. Hba1c or UACR approach), and 17.5 (95% CI 10.0 to 23.0) (P < 0.001 vs. Hba1c or UACR approach, and P = 0.54 versus clinical risk model) events prevented per 1000 patients treated for 5 years, respectively. For the composite of heart failure outcome, the NNT to prevent one event were 51.0 (95% CI 34.2 to 125.3), 47.0 (95% CI 31.0 to 115.5), 39.0 (95% CI 26.1 to 95.7), and 39.0 (95% CI 25.7 to 94.2), respectively (Fig. 5B). This corresponded to 16.0 (95% CI 6.5 to 24.0), 16.9 (95% CI 6.9 to 25.5), 21.0 (95% CI 8.5 to 31.4) (P < 0.01 vs. Hba1c or UACR approach), and 21.3 (95% CI 8.7 to 31.9) (P < 0.01 vs. Hba1c or UACR approach, and P = 0.85 versus clinical risk model) events prevented per 1000 patients treated for 5 years, respectively. The NNT to prevent one composite kidney or heart failure outcomes among patients with albuminuria are shown in Additional file 1: Figs. S1B and S2B).

## Discussion

Patients with type 2 diabetes are at increased risk of developing kidney and heart failure complications. We demonstrated that a risk model consisting of clinically accessible patient information accurately predicted the long-term risk of these events. We furthermore showed that novel biomarkers only modestly increased the predictive performance of the clinical model. Finally, we demonstrated a large variation in the predicted 5-year benefit of the SGLT2 inhibitor canagliflozin for the composite kidney and heart failure or CV death outcomes and that treating patients with type 2 diabetes with the SGLT2 inhibitor canagliflozin according to a multivariable risk prediction model is more effective than a treatment strategy based on HbA1c or UACR alone. These data support the use of a multivariable risk assessment strategy to guide SGLT2 inhibitor initiation.

Our multivariable risk models accurately predicted the kidney outcomes. The predictors in the risk models were similar to other previously developed risk models [22]. In keeping with previous studies, UACR was the strongest predictor in the model [23, 24]. In our study, baseline HbA1c did not predict the long-term kidney outcome and was therefore excluded from the final model. Since HbA1c was targeted during the CANVAS trial this may have led to the exclusion of HbA1c from the kidney risk prediction model. Despite UACR being a strong predictor of kidney failure, initiation of canagliflozin based on an estimated risk-based approach was more effective suggesting that other risk markers contribute to the risk of kidney failure as well. Novel biomarkers representing inflammatory and fibrosis pathways were independently associated with the kidney and heart failure outcomes highlighting the well-known involvement of inflammation in the cardio-renal pathophysiology [25–27]. Nevertheless, there was no clear incremental predictive performance of these novel biomarkers on top of clinical predictors in our study.

The clinical model predicting kidney outcomes also predicted heart failure. This illustrates the close interaction between kidney and heart failure as demonstrated in previous studies as well [28, 29]. In a cohort of 4,500,000 individuals, the use of a kidney risk model was shown to improve the 10-year heart failure risk prediction, highlighting the important interplay between the two conditions [30]. Moreover, in the Thrombolysis in Myocardial Infarction (TIMI) Risk Score for heart failure in patients with type 2 diabetes, eGFR and UACR were among the top 5 predictors of heart failure risk in patients with type 2 diabetes of 25 candidate risk markers, while the other 3 predictors were history of heart failure, history of atrial fibrillation, and the presence of coronary artery disease [31]. Despite the fact that heart failure is common in patients with diabetic kidney disease, a “risk-treatment” paradox exists such that proven effective heart failure drugs are less frequently prescribed when kidney function declines [32]. These data underscore the need for implementation of evidence-based therapies in patients with diabetic kidney disease to prevent heart failure. Further evidence about the interplay between the heart and the kidney stems from biomarker studies demonstrating that biomarkers of kidney disease progression, such as the ones identified in our study, TNFR-1, KIM-1, IL-6, MMP-7, also predict the composite heart failure outcome [33–35].

Using the multivariable risk models, we demonstrated a large variation in the predicted 5-year risk for the composite kidney and the composite heart failure outcome alongside a large variation in absolute treatment effects. For example, patients with 5-year predicted composite kidney risk greater than 5% were found in a relatively small proportion. The likely explanation is that the CANVAS program included few patients with CKD and there were only 28% of the participants who had UACR ≥ 30 mg/g. Nevertheless, we observed a consistent findings in those with albuminuria where the use of multivariable risk-based approach results in more events prevented and lower NNT as compared to a treatment strategy guided by HbA1c or UACR alone. In our study, no heterogeneity of treatment effect was observed indicating that the relative treatment effect of canagliflozin is consistent across a range of predicted baseline risk levels.

Our results demonstrating that a multivariable risk-strategy is more efficient to guide SGLT2 initiation than a strategy based on a single risk marker (i.e., HbA1c or albuminuria) are consistent with previous studies showing the micro- and macrovascular complications in patients with type 2 diabetes are determined by multiple risk markers, and support the measurement of biomarker panels to adequately capture individual risk and guide individualized treatment decisions [36, 37]. The utility of risk-based approaches to optimize and individualize therapy decisions have been demonstrated for other cardio-renal drugs [38, 39]. Further support of the monitoring and targeting of multiple risk factors in patients with type 2 diabetes stems from randomized controlled trials demonstrating that intensive multifactorial risk marker management significantly reduces the risk of renal and CV complications as compared to conventional risk marker management [40, 41].

Several limitations should be considered when interpreting the findings. Firstly, there were only few patients who had HbA1c ≥ 10.5% (n = 54), and albuminuria (n = 1037) at baseline, thus the risk prediction and ARR estimation in this group are based upon small samples. Secondly, we note that the model was developed and tested in the same trial and was not externally validated. However, the model was internally validated and was corrected for optimism. Our main aim was not to generate a model for external use but to demonstrate the utility of a multivariable model with known predictors versus HbA1c and UACR alone for the initiation of SGLT2 treatment. Thirdly, we used NNT as a metrics of risk-benefit assessment in this study. Future studies could use other measures of effectiveness including assessment of cost-effectiveness for the different strategies. Finally, it is important to note that although SGLT2 inhibitors have shown strong beneficial effects on the kidney and heart failure outcomes, canagliflozin did not statistically significantly decrease the risk of all-cause mortality in the CANVAS program. Therefore we did not include all-cause mortality in the current analyses.

## Conclusion

In conclusion, this post-hoc analysis of the CANVAS trial suggests that the use of a clinical risk-based assessment is more efficient to guide the initiation of SGLT2 inhibitors for patients with type 2 diabetes instead of using HbA1c or UACR alone. This would result in the prevention of more kidney and heart failure event for the same number of patients treated compared to the HbA1c or UACR guided initiation of SGLT2 inhibitors across a broad range of potential thresholds to initiate treatment. Validation in an independent study would support clinical implementation.

## Supplementary Information

**Additional file 1**: **Box S1. **Prediction of the effect ofcanagliflozin on the composite kidney risk for individual patients with type 2diabetes in the CANVAS trial according to clinical and novel markers model.  **Table S1. **Hazard ratios and P-for-interactions between predictedbaseline risk and treatment variable for both the composite kidney and thecomposite of heart failure or cardiovascular death outcomes. **Figure S1. **(A) The number of events prevented in patientswith albuminuria (UACR ≥30mg/g, n=1037) based on the HbA1c (red line), urinary-albumin-creatinineratio (UACR) (purple line), clinical markers (green line), or clinical andnovel biomarkers (blue line) strategy for the composite kidney outcome(defined as the composite of sustained 40% decline of eGFR, end-stage kidneydisease with eGFR <15 mL/min/1.73m², or need for dialysis or kidneytransplantation, or kidney death),and C-statistics obtained for the respective model. Numbers at each curve arespecific HbA1c, UACR cut-offs or based on 5th to 95th percentiles of predicted5-year risk at specific treatment threshold. (B) The number needed to treat inpatients with albuminuria (UACR ≥30mg/g, n=1037) in order to avoid onecomposite heart failure outcome according to the HbA1c (red line), UACR (purpleline), clinical markers (green line) or the clinical and novel markers (blueline) strategies are shown in the same figure. The intersection points at thevertical dashed-lines indicate the corresponding events prevented and thenumber needed to treat for the different strategies for the same number ofpatients treated with UACR ≥30mg/g and UACR≥300mg/g (n = 214). **Figure S2. **(A)The number of events prevented in patients with albuminuria (UACR ≥30mg/g,n=1037) based on the HbA1c (red line), urinary-albumin-creatinine ratio (UACR)(purple line), clinical markers (green line), or clinical and novel biomarkers(blue line) strategy for the composite heart failure outcome (defined as heartfailure hospitalization or CV death) outcome, and C-statistics obtained for therespective model. Numbers at each curve are specific HbA1c, UACR cut-offs orbased on 5th to 95th percentiles of predicted 5-year risk at specific treatmentthreshold. (B) The number needed to treat in patients with albuminuria (UACR≥30mg/g, n=1037) in order to avoid one composite heart failure outcomeaccording to the HbA1c (red line), UACR (purple line), clinical markers (greenline) or the clinical and novel markers (blue line) strategies are shown in thesame figure. The intersection points at the vertical dashed-lines indicate thecorresponding events prevented and the number needed to treat for the differentstrategies for the same number of patients treated with UACR ≥30mg/g and UACR≥300mg/g(n = 214).

## Data Availability

The datasets used and/or analyzed during the current study are available from the corresponding author on reasonable request.

## Abbreviations

SGLT2: Sodium glucose cotransporter-2
CV: Cardiovascular
CKD: Chronic kidney disease
TNFR: Tumor necrosis factor receptor
KIM: Kidney injury molecule
GDF: Growth differentiation factor
IL: Interleukins
MMP: Matrix metallopeptidase
uMCP: Urinary monocyte chemoattractant protein
uEGF: Urinary epidermal growth factor
BP: blood pressure
HbA1c: Glycated hemoglobin
UACR: Urinary albumin to creatinine ratio
HDL: High-density-lipoprotein cholesterol
LDL: Low-density-lipoprotein cholesterol
eGFR: Estimated glomerular filtration rate
CKD-EPI: Chronic kidney disease epidemiology collaboration
Cr: Creatinine
OTR: On-treatment risk
NNT: Number needed to treat
