# Supplementary material for: Initiation of the SGLT2 inhibitor canagliflozin to prevent kidney and heart failure outcomes guided by HbA1c, albuminuria, and predicted risk of kidney failure
Source: Cardiovasc Diabetol. 2022 Sep 23;21:194. doi: 10.1186/s12933-022-01619-0 (PMC9508745; doi:10.1186/s12933-022-01619-0)
Supplement: Supplementary file 1 — Additional file 1: Box S1. Prediction of the effect ofcanagliflozin on the composite kidney risk for individual patients with type 2diabetes in the CANVAS trial according to clinical and novel markers model. Table S1. Hazard ratios and P-for-interactions between predictedbaseline risk and treatment variable for both the composite kidney and thecomposite of heart failure or cardiovascular death outcomes. Figure S1. (A) The number of events prevented in patientswith albuminuria (UACR ≥30mg/g, n=1037) based on the HbA1c (red line), urinary-albumin-creatinineratio (UACR) (purple line), clinical markers (green line), or clinical andnovel biomarkers (blue line) strategy for the composite kidney outcome(defined as the composite of sustained 40% decline of eGFR, end-stage kidneydisease with eGFR <15 mL/min/1.73m², or need for dialysis or kidneytransplantation, or kidney death),and C-statistics obtained for the respective model. Numbers at each curve arespecific HbA1c, UACR cut-offs or based on 5th to 95th percentiles of predicted5-year risk at specific treatment threshold. (B) The number needed to treat inpatients with albuminuria (UACR ≥30mg/g, n=1037) in order to avoid onecomposite heart failure outcome according to the HbA1c (red line), UACR (purpleline), clinical markers (green line) or the clinical and novel markers (blueline) strategies are shown in the same figure. The intersection points at thevertical dashed-lines indicate the corresponding events prevented and thenumber needed to treat for the different strategies for the same number ofpatients treated with UACR ≥30mg/g and UACR≥300mg/g (n = 214). Figure S2. (A)The number of events prevented in patients with albuminuria (UACR ≥30mg/g,n=1037) based on the HbA1c (red line), urinary-albumin-creatinine ratio (UACR)(purple line), clinical markers (green line), or clinical and novel biomarkers(blue line) strategy for the composite heart failure outcome (defined as heartfailure hospitalization or CV death) outcome, and C [file 12933_2022_1619_MOESM1_ESM.docx]

**Initiation of the SGLT2 inhibitor canagliflozin to prevent kidney and cardiovascular outcomes guided by HbA1c and predicted risk of kidney failure**

Sok Cin Tye^1^, Niels Jongs^1^, Steven G Coca^2^, Johan Sundström^3,4^, Clare Arnott^4,5^, Bruce Neal^4^, Vlado Perkovic^4^, Kenneth W Mahaffey^6^, Priya Vart^1^, Hiddo. J. L. Heerspink^1, 4^.

^1^Department of Clinical Pharmacy and Pharmacology, University of Groningen, University Medical Center Groningen, Groningen, The Netherlands.

^2^Division of Nephrology, Department of Medicine, Icahn School of Medicine at Mount Sinai, New York, New York, USA.

^3^Department of Medical Sciences, Uppsala University, Uppsala, Sweden

^4^The George Institute for Global Health, UNSW Sydney, Sydney, NSW, Australia

^5^Department of Cardiology, Royal Prince Alfred Hospital, Sydney, Australia.

^6^Stanford Center for Clinical Research, Department of Medicine, Stanford University, Stanford, CA, USA

**Additional file 1**

**Box S1. Prediction of the effect of canagliflozin on the composite kidney risk for individual patients with type 2 diabetes in the CANVAS trial according to clinical and novel markers model.**

| **Prediction model for the estimation of ARR of canagliflozin on the composite kidney risk in type 2 diabetes patients** |
| --- |
| **5-year composite kidney risk (%)=** 1- 0.9858^exp(linear predictor)*100% |
| **Linear predictor for clinical marker model=** (0.021342*age in years)+ (0.711029*previous cardiovascular history)+ (0.007996*systolic blood pressure)+(0.545536* log-urinary-albumin-creatinine ratio)+(-0.016713*hemoglobin)+(0.008591*body weight)+(-0.094547*albumin)+(0.004377*eGFR)+0.422  **Linear predictor for clinical and novel marker model=** (0.022539*age in years)+ (0.749525*previous cardiovascular history)+ (0.006723*systolic blood pressure)+( 0.406400* log-urinary-albumin-creatinine ratio)+(-0.012103*hemoglobin)+( 0.004557*body weight)+( -0.057843 *albumin)+(0.023357*eGFR)+(1.219490* log-TNFR1)+(0.428380*log-MMP7)+(0.254804*log-KIM1)+(0.220902*log-IL6)+0.4449 |
| **On-treatment risk (%)=** 5-year kidney risk (%)*0.60 [actual HR observed in the trial] |
| **Individual absolute risk reduction (ARR, %)=** 5-year predicted kidney risk (%) – On-treatment risk (OTR) |

**Footnote:** TNFR, tumor necrosis factor receptor; KIM, kidney injury molecule; MMP, matrix metallopeptidase; IL, interleukin. The estimated glomerular filtration rate (eGFR) was calculated using the Chronic Kidney Disease Epidemiology Collaboration (CKD-EPI) formula according to the Chronic Kidney Disease Epidemiology Collaboration formula as per the CANVAS trial protocol.

**Table S1.** Hazard ratios and P-for-interactions between predicted baseline risk and treatment variable for both the composite kidney and the composite of heart failure or cardiovascular death outcomes.

| **Outcomes** | **Model** | **Predicted risk categories** | **Main effect** | | **P _for interaction_** |
| --- | --- | --- | --- | --- | --- |
|  |  |  | **Hazard ratios** | **95% CI** |  |
| Composite kidney | Clinical markers | Low risk (0 to <5%) | **Reference** | | 0.106 |
|  |  | Intermediate risk (5 to <15%) | 2.02 | 0.88 to 4.63 |  |
|  |  | High risk (≥15%) | 0.83 | 0.37 to 1.84 |  |
|  | Clinical and novel markers | Low risk (0 to <5%) | **Reference** | | 0.913 |
|  |  | Intermediate risk (5 to <15%) | 1.14 | 0.52 to 2.53 |  |
|  |  | High risk (≥15%) | 0.96 | 0.43 to 2.14 |  |
| Composite heart failure | Clinical markers | Low risk (0 to <5%) | **Reference** | | 0.561 |
|  |  | Intermediate risk (5 to <15%) | 1.14 | 0.55 to 2.37 |  |
|  |  | High risk (≥15%) | 0.89 | 0.42 to 1.88 |  |
|  | Clinical and novel markers | Low risk (0 to <5%) | **Reference** | | 0.750 |
|  |  | Intermediate risk (5 to <15%) | 0.83 | 0.42 to 1.64 |  |
|  |  | High risk (≥15%) | 0.97 | 0.49 to 1.92 |  |


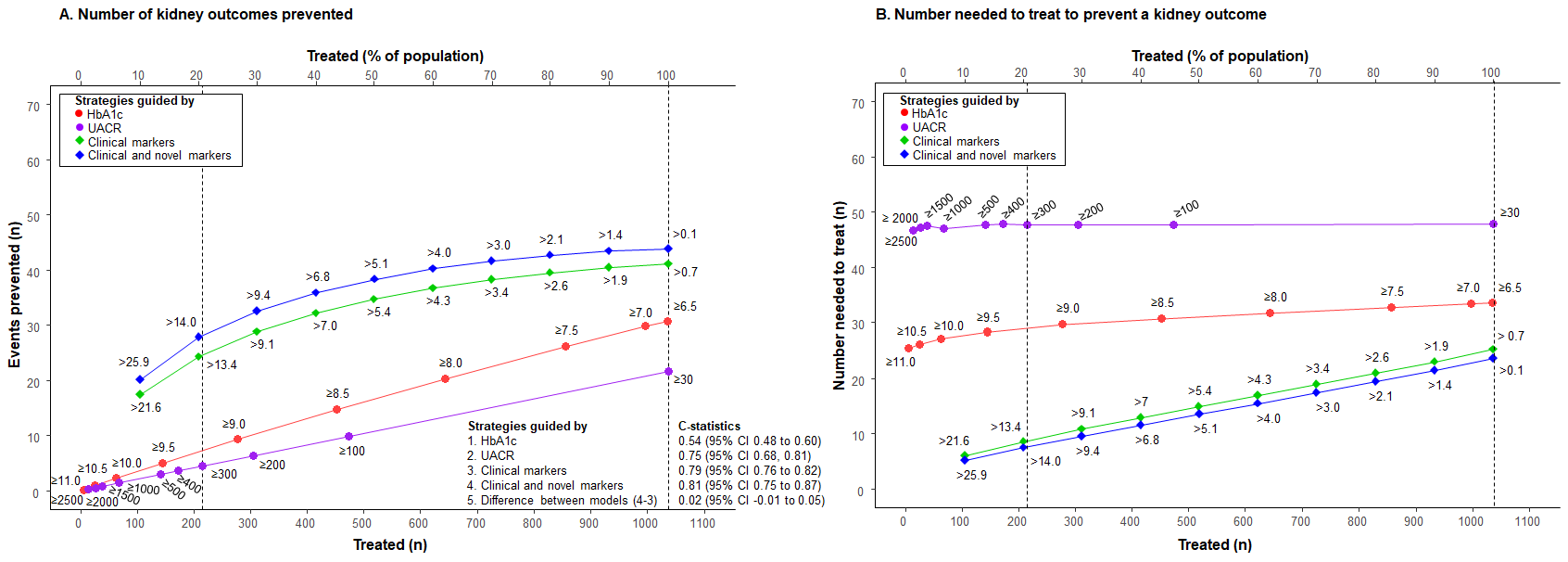


**Figure S1.** (A) The number of events prevented in patients with albuminuria (UACR ≥30mg/g, n=1037) based on the HbA1c (red line), urinary-albumin-creatinine ratio (UACR) (purple line), clinical markers (green line), or clinical and novel biomarkers (blue line) strategy for the composite kidney outcome (defined as the composite of sustained 40% decline of eGFR, end-stage kidney disease with eGFR <15 mL/min/1.73m², or need for dialysis or kidney transplantation, or kidney death), and C-statistics obtained for the respective model. Numbers at each curve are specific HbA1c, UACR cut-offs or based on 5th to 95th percentiles of predicted 5-year risk at specific treatment threshold. (B) The number needed to treat in patients with albuminuria (UACR ≥30mg/g, n=1037) in order to avoid one composite heart failure outcome according to the HbA1c (red line), UACR (purple line), clinical markers (green line) or the clinical and novel markers (blue line) strategies are shown in the same figure. The intersection points at the vertical dashed-lines indicate the corresponding events prevented and the number needed to treat for the different strategies for the same number of patients treated with UACR ≥30mg/g and UACR≥300mg/g (n = 214).


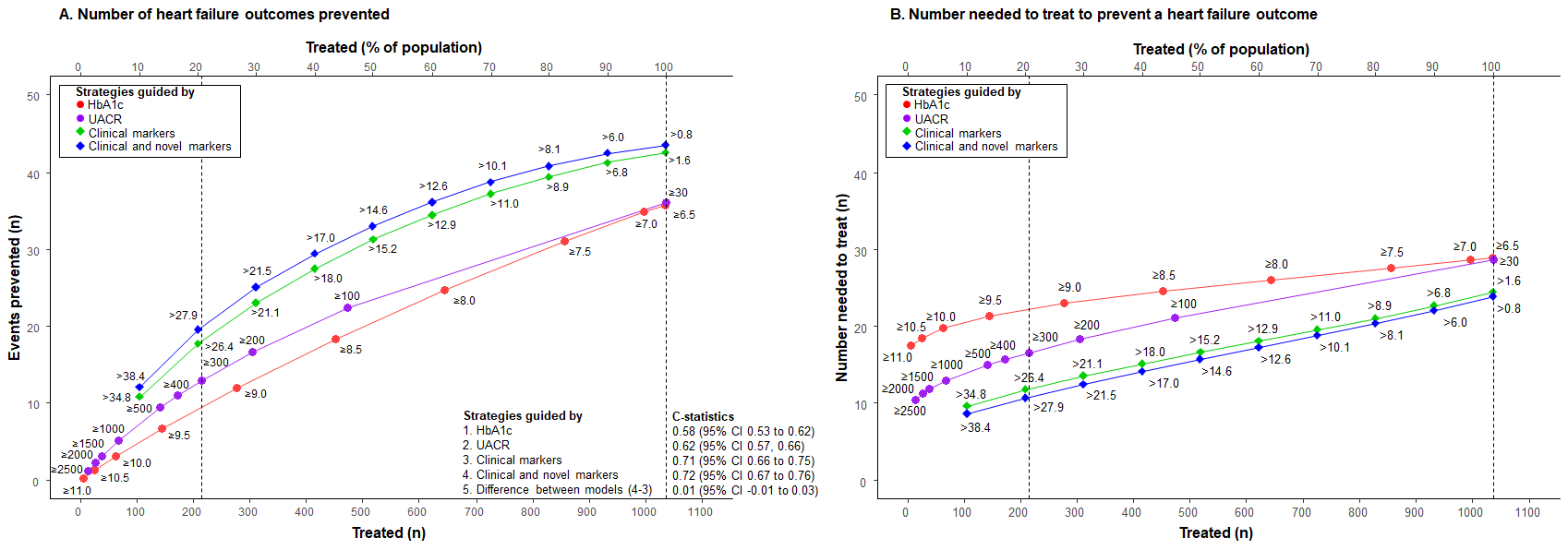


**Figure S2.** (A) The number of events prevented in patients with albuminuria (UACR ≥30mg/g, n=1037) based on the HbA1c (red line), urinary-albumin-creatinine ratio (UACR) (purple line), clinical markers (green line), or clinical and novel biomarkers (blue line) strategy for the composite heart failure outcome (defined as heart failure hospitalization or CV death) outcome, and C-statistics obtained for the respective model. Numbers at each curve are specific HbA1c, UACR cut-offs or based on 5th to 95th percentiles of predicted 5-year risk at specific treatment threshold. (B) The number needed to treat in patients with albuminuria (UACR ≥30mg/g, n=1037) in order to avoid one composite heart failure outcome according to the HbA1c (red line), UACR (purple line), clinical markers (green line) or the clinical and novel markers (blue line) strategies are shown in the same figure. The intersection points at the vertical dashed-lines indicate the corresponding events prevented and the number needed to treat for the different strategies for the same number of patients treated with UACR ≥30mg/g and UACR≥300mg/g (n = 214).
